# Hepatocellular Carcinoma: Current Drug Therapeutic Status, Advances and Challenges

**DOI:** 10.3390/cancers16081582

**Published:** 2024-04-20

**Authors:** Shunzhen Zheng, Siew Wee Chan, Fei Liu, Jun Liu, Pierce Kah Hoe Chow, Han Chong Toh, Wanjin Hong

**Affiliations:** 1 Key Laboratory of Biopharmaceuticals, Postdoctoral Scientific Research Workstation, Shandong Academy of Pharmaceutical Science, Jinan 250098, China; liufei@sdaps.cn; 2 Institute of Molecular and Cell Biology (IMCB), Agency for Science, Technology and Research (A*STAR), 61 Biopolis Drive, Singapore 138673, Singapore; mcbcsw@imcb.a-star.edu.sg (S.W.C.); mcbhwj@imcb.a-star.edu.sg (W.H.); 3 Department of Hepatobiliary Surgery, Shandong Provincial Hospital Affiliated to Shandong First Medical University, Jinan 250021, China; dr_liujun1967@126.com; 4 Division of Surgery and Surgical Oncology, National Cancer Centre, Singapore 169610, Singapore; pierce.chow@duke-nus.edu.sg; 5 Academic Clinical Programme for Surgery, Duke-NUS Medical School, Singapore 169857, Singapore; 6 Division of Medical Oncology, National Cancer Centre Singapore, Singapore 168583, Singapore; toh.han.chong@singhealth.com.sg

**Keywords:** hepatocellular carcinoma, genetic alterations, molecular targeted therapy, immunotherapy

## Abstract

**Simple Summary:**

Hepatocellular carcinoma is the second leading cause of cancer-related deaths and the seventh most common cancer worldwide. Although there have been rapid developments in the treatment of hepatocellular carcinoma over the past decade, the incidence and mortality rates of hepatocellular carcinoma remain challenging. Only about 30% of patients can be treated with curative methods, while over 50% of patients require systemic treatment to prolong survival, with a limited benefit. Molecular targeted therapy and immunotherapy have brought about a revolution in hepatocellular carcinoma systemic treatment. Nevertheless, the treatment of hepatocellular carcinoma is still a challenge due to significant drug resistance, tumor heterogeneity, lack of druggable mutation targets, and lack of effective biomarkers. To improve outcomes of hepatocellular carcinoma patients, we need to gain a deeper understanding of the hepatocellular carcinoma genome and explore more combination treatment regimens.

**Abstract:**

Hepatocellular carcinoma (HCC) is the most common form of liver cancer, accounting for ~90% of liver neoplasms. It is the second leading cause of cancer-related deaths and the seventh most common cancer worldwide. Although there have been rapid developments in the treatment of HCC over the past decade, the incidence and mortality rates of HCC remain a challenge. With the widespread use of the hepatitis B vaccine and antiviral therapy, the etiology of HCC is shifting more toward metabolic-associated steatohepatitis (MASH). Early-stage HCC can be treated with potentially curative strategies such as surgical resection, liver transplantation, and radiofrequency ablation, improving long-term survival. However, most HCC patients, when diagnosed, are already in the intermediate or advanced stages. Molecular targeted therapy, followed by immune checkpoint inhibitor immunotherapy, has been a revolution in HCC systemic treatment. Systemic treatment of HCC especially for patients with compromised liver function is still a challenge due to a significant resistance to immune checkpoint blockade, tumor heterogeneity, lack of oncogenic addiction, and lack of effective predictive and therapeutic biomarkers.

## 1. Introduction

**Figure 1:**
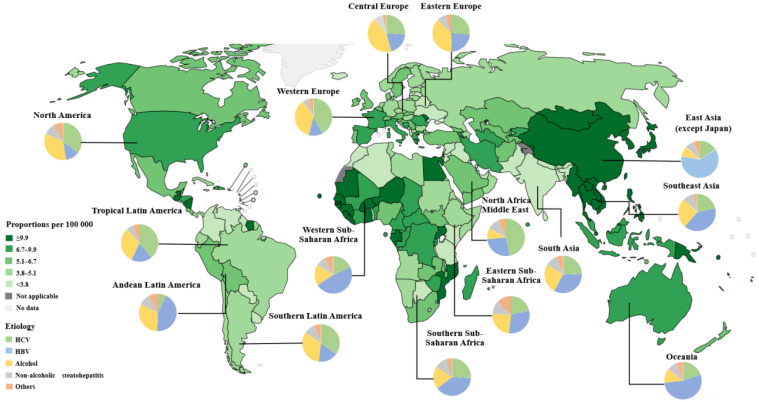
The estimated number of prevalent cases (5 years) of liver cancer, according to geographical area and etiology, in 2020. Hepatitis B virus (HBV) is the major etiological factor in Asia (except in Japan, where HCV is the major risk factor for liver cancer). HCV is the dominant causative factor in North America and Western Europe. Excessive alcohol intake is an etiological factor in Central and Eastern Europe. The prevalence of metabolic risk factors for HCC, including metabolic syndrome, obesity, type 2 diabetes, and MAFLD/MASH, is increasing worldwide. Estimated number of prevalent cases (5 years) as a proportion in 2020, liver, both sexes, all ages, Copyright (2020). (https://gco.iarc.who.int/en) (accessed on 10 March 2023). Etiology data from [6].

Primary liver cancer is the seventh most common cancer and the second leading cause of cancer-related deaths in the world [1]. The World Health Organization (WHO) estimates that liver cancer will cause more than one million deaths in 2030 [2]. HCC is the most common form of primary liver cancer, accounting for ~90% of cases [3]. Asia and Africa have the highest incidence of HCC in the world. Due to a higher incidence of hepatitis B virus infection, as well as having one of the largest populations in the world, China has the highest number of HCC cases, accounting for about half of global cases [4] (Figure 1). HCC is the fifth most common cancer and the second leading cause of cancer-related deaths in China [5].

Currently, hepatitis B virus (HBV) and hepatitis C virus (HCV) remain the most significant global risk factors for HCC [7]. However, with the promotion of universal neonatal hepatitis vaccination and improved access to antiviral therapy for chronic hepatitis infections, the incidence of virus-related HCC has been on the decline, especially in countries with a high incidence of viral hepatitis. Taking HBV as an example, the infection rate of HBV in the United States and Western Europe is 0.1%~2%. In Japan and Mediterranean countries, the infection rate is 2%~8%, while in most African and Asian countries it is 8%~20%. In 1992, the HBV surface antigen (HBsAg)-positive rate among the Chinese population was 9.8%. Subsequently, the government issued and implemented a universal HBV vaccination program for newborn babies. The HBsAg-positive rate in China decreased from 9.8% in 1992 to 7.2% in 2006, with the HBsAg-positive rate in children under 10 years of age dropping to 1.5% [8]. Furthermore, in children under 5 years old, the HBsAg-positive rate dropped to less than 0.4% in 2019 [9]. It is evident that the HBV vaccine has played a significant role in the prevention and control of hepatitis in China; as a result, the proportion of Chinese HCC patients in the world decreased from 55.0% in 2008 to 45.3% in 2020 [5].

However, the prevalence of metabolic risk factors for HCC, including metabolic syndrome, obesity, type 2 diabetes, and metabolic dysfunction-associated steatotic liver disease (MASLD), is increasing and becoming the main cause of HCC growth, especially in Western countries [10]. In addition, traditional factors for HCC, such as excessive alcohol consumption, aflatoxin contamination, and smoking, remain significant and cannot be ignored in the incidence of HCC. Because almost all HCC risk factors are theoretically preventable, the prevention of high-risk factors should be a key strategy for reducing HCC incidence and improving HCC prognosis.

The overall prognosis of HCC is poor, and treatment outcomes are still unsatisfactory. The data show that in 2018, the incidence and mortality of HCC were roughly equivalent [11]. HCC presents different prognoses and drug responses based on etiology. Generally, the prognosis of HBV-related HCC is poorer. Possible reasons for this include that HBV can integrate into the genome of patients, and compared to HCV, antiviral therapy for HBV is less effective. Furthermore, HBV-related HCC is usually accompanied by more severe molecular alterations, such as p53 mutations, leading to more malignant molecular biology behavior [12].

Due to inefficient surveillance, metabolic-related HCC tends to be diagnosed later, and this type of HCC tends to respond more poorly to immunotherapy in the advanced but not adjuvant setting, which means that a potential improvement in prognosis is able to be expected. The molecular pathogenesis has remained much less clear than that of viral hepatitis; to address this problem, a series of studies are underway, including the PLANet and ELEGANCE programs in Singapore [13].

The prognosis and treatment strategy of Barcelona Clinic Liver Cancer (BCLC) are well known, and management guidelines for HCC have been established in many regions of the world. Also, the BCLC system is useful to compare the outcomes of different therapies and is frequently used in clinical trials worldwide. In the early stages, curative methods such as surgical resection, liver transplantation, and radiofrequency ablation can significantly prolong the survival of patients. However, due to the poor efficiency of screening and surveillance strategies, many patients miss the opportunity to receive curative treatments because of late tumor staging at the time of diagnosis, resulting in limited non-curative methods. Consequently, they have to resort to loco-regional therapy or systemic therapy to prolong their lives. [14]. Compared to the slow progress made in surgical treatment, systemic therapy has undergone rapid development, both in neoadjuvant therapy for early intermediate HCC and adjuvant therapy for advanced HCC, over the past decade [15]. Since the Food and Drug Administration (FDA) approved Sorafenib for the treatment of HCC in 2007, molecular-targeted therapy and immunotherapy (such as kinase inhibitors (KIs), anti-angiogenic agents (AAs), and immune checkpoint inhibitors (ICIs)) have brought about revolutionary changes [16]. However, in the real world, the efficiency of targeted and immunotherapy drugs still cannot meet clinical needs, manifesting high primary resistance rates, adaptive resistance rates, and acquired resistance rates. Additionally, the lack of druggable targets with high mutation rates, the absence of effective molecular biomarkers for patient stratification and treatment guidance, as well as a lack of consensus on drug combination regimens have limited the effectiveness of targeted therapy and immunotherapy [17]. In recent years, numerous clinical trials have provided evidence for the rational use of targeted and immunotherapeutic agents, greatly improving the efficacy of systemic treatment of HCC.

This article discusses the current status, advances, and challenges of HCC clinical treatment based on the molecular genetic alterations of HCC. The progress undertaken in systemic regimens will be the focus of the discussion in this review.

## 2. Genetic Alterations and Signaling Pathways in HCC

**Figure 2:**
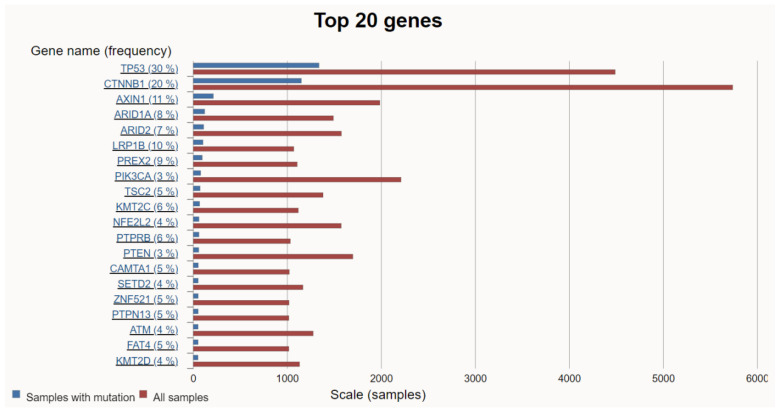
The top 20 genetic mutations in HCC. (https://cancer.sanger.ac.uk/cosmic/browse/tissue?wgs=off&sn=liver&ss=all&hn=carcinoma&sh=hepatocellular_carcinoma&in=t&src=tissue&all_data=n) (accessed on 5 February 2024).

HCC is a highly heterogeneous tumor resulting from the accumulation of various genetic mutations. Among them, mutations that promote HCC proliferation and metabolism are defined as driver mutations, while other mutations that confer no selective growth advantage are defined as passenger mutations [18]. Over the past decade, the accumulation of high-throughput analysis data from numerous samples has facilitated a deeper understanding of the molecular pathogenesis of HCC. As a result, some alterations, such as TERT promoter, TP53, CTNNB1, AXIN1, ARID1A, ARID2, LRP1B, TSC2, PTEN, MYC, and JAK1, were revealed as common HCC driver mutations [19,20]. Searching the COSMIC site for HCC revealed the top 20 genetic mutations in HCC (Figure 2).

A study based on the European HCC population provided a relatively complete description of the genetic alterations associated with HCC. The study identified 11 pathways altered with a mutation rate of ≥5%, including TERT promoter mutations activating telomerase expression (60%), CTNNB1 (WNT/β-catenin) (54%), PI3K/AKT/mTOR (51%), TP53 (49%), MAPK (43%), the genetic alterations involved in hepatic differentiation (34%), epigenetic regulation (32%), chromatin remodeling (28%), oxidative stress (12%), IL-6/JAK-STAT (9%), and TGF-β (5%) [21]. In this study, alcohol-related HCC was significantly enriched in CTNNB1 (WNT/β-catenin), TERT, CDKN2A, SMARCA2, and HGF alterations. HBV-associated HCC is frequently mutated in TP53, and IL-6/JAK-STAT mutations have been exclusively found in HCCs of unknown etiology [21]. It should be noted that this study was based on a European HCC population, meaning that it may not totally reflect the real situation in other regions of the world due to the different risk factors causing HCC [22,23].

In addition, several studies have identified genetic mutations in HCC which correlate with tumor stages. For example, mutations of TERT were early events in HCC. The frequency of CTNNB1 (WNT/β-catenin) and TP53 mutations significantly increased in those with advanced tumors, while amplification of the FGF19/CNND1 locus was mainly observed in those with HCC with poor prognosis [21,24,25].

However, China accounts for half of the world’s HCC cases, and 90% of patients are associated with HBV infection. Recently, through the Chinese Liver Cancer Atlas (CLCA) project, deep whole genome sequencing was performed on 494 HCC-HBV in Chinese individuals, and 23 candidate coding cancer drivers and 31 candidate non-coding drivers were identified. Compared with the Pan-Cancer Analysis of Whole Genomes (PCAWG)-HCC cohort, the CLCA cohort had higher proportions of HBV infection (94.5% versus 30.6%) and Edmondson-Steiner grades 3 and 4 (85.6% versus 12.1%), while it had lower proportions of hepatitis C virus (HCV) infection (2.6% versus 55.6%), alcohol drinking (26.7% versus 58.1%), and smoking (36.8% versus 53.6%). TP53 mutations were significantly more frequent in CLCA than in PCAWG-HCC or TCGA-HCC, while CTNNB1 mutations were mutually exclusive with either TP53 or AXIN1 mutations, which is consistent with HCC in European individuals [21]. Notably, the CLCA found that HBV integrations could take the form of extrachromosomal circular DNA and that characterized catastrophic events could occur in the late stage of HCC, including chromotropic thripsis, chromotropic plexy, and kataegis [26]. The CLCA, based on deep whole genome sequencing, identified six coding cancer drivers and 28 non-coding drivers that were previously unreported for HCC, suggesting that our understanding of the HCC genome is still very limited.

HCC can be classified into two subtypes based on genomic profiling: proliferative and nonproliferative [3,19,27,28]. Proliferative HCC is characterized by the activation of signaling cascades involved in cell proliferation, the enrichment of poor prognostic signals, and association with the clinical features of an aggressive tumor and poor prognosis. Activated signaling pathways include AKT/mTOR, MET, TGFβ, IGF, and RAS/MAPK. Clinically, proliferative HCC patients have a higher incidence of invasive tumors, higher levels of alpha-fetoprotein [29], poor histological cell differentiation, and frequent vascular invasion [30]. HBV-related HCC mainly belongs to this subtype. Patients with such tumors have a higher risk of recurrence after resection and a shorter survival period.

In nonproliferative HCC, activation of the WNT/β-catenin signaling pathway is relatively high [31], and the tumor transcriptome is more similar to normal liver physiology. Clinically, such tumors exhibit a less aggressive phenotype, including better histological differentiation, lower alpha-fetoprotein, and lack of enrichment in adverse prognostic features [32]. HCV and alcohol-related HCC are more common in this subtype [33].

In this section, we discuss some of the most common mutations detected in HCC.

### 2.1. Telomerase Reverse Transcriptase

TERT mutations are the most common molecular alteration in HCC, along with the entire process of hepatocyte transformation from cirrhosis to HCC [28]. This process accompanies a transformation of liver lesion blood supply from the portal vein to the hepatic artery, as well as an increase in invasiveness and metastatic potential [34,35].

In the human liver, telomerase is not expressed in mature hepatocytes. Cirrhotic tissues exhibit telomere shortening, accompanied by replicative senescence, while telomerase is reactivated in more than 90% of HCC cases. Lineage-specific TERT mutations and telomerase reactivation are key events in the malignant transformation of hepatocytes [36,37]. The mechanisms of telomerase reactivation include somatic TERT promoter mutations (54%–60%) and TERT amplification (5%–6%), as well as HBV inserted into the TERT promoter (10%–15%) [19,38], and these mechanisms are mutually exclusive. In addition, TERT promoter mutations often work synergistically with CTNNB1 (WNT/β-catenin) mutations in liver tumorigenesis. TERT promoter mutations appear in 6% of low-grade dysplastic nodules (LGDNs) and 19% of high-grade dysplastic nodules (HGDNs), and the frequency of TERT promoter mutations dramatically increases in early HCC (61%) and remains high in advanced and terminal-stage HCC [39].

### 2.2. TP53

Studies have shown that about 49% of HCCs present TP53 mutations [40,41]. Genomic aberrations in the p53 pathway are the most common abnormalities in various types of cancer and are generally associated with HBV infection, higher histological grades, stronger vascular invasion ability, and poorer prognosis in HCC. The TP53 gene regulates a variety of biological processes. Aflatoxin B1 (AFB1) exposure-related HCC is confirmed to be related to the TP53 R249S mutation [42]. Additionally, the expression of VEGFA is also regulated by TP53 [43,44].

TP53 mutations are more frequent in advanced-stage HCC. The TP53 mutation rate in BCLC C tumors is 35%, while it is only 15.5%–17.3% in BCLC stage 0-B tumors [45]. Current research also indicates that many HCC cases without TP53 mutations present TP53 inactivation, and TP53-inactivated HCC is also accompanied by changes in a series of protein expressions in the p53 pathway, thereby promoting the occurrence and development of HCC [45].

### 2.3. CTNNB1 (WNT/β-Catenin)

The WNT/β-catenin pathway plays a key role in liver physiological embryogenesis, differentiation, and metabolic control; it is one of the most frequent oncogenic mutations in HCC [31]. This pathway is frequently activated in HCC by activating CTNNB1 mutations (11%–37%), resulting in their increased stability and nuclear translocation to drive oncogenic transcription [46]. Inactivating AXIN1 mutations (5%–35%) and APC mutations (1%–2%) also lead to β-catenin activation [19,47].

### 2.4. PI3K/AKT/mTOR and RAS/RAF/MAPK

The PI3K/AKT/mTOR and RAS/RAF/MAPK pathways are downstream of tyrosine kinase receptors and are involved in cell growth, proliferation, and survival [48]. Activating mutations of FGF19/CCND1 and PIK3CA, inactivating mutations of TSC1 or TSC2, and the homozygous deletion of PTEN can all activate the PI3K/AKT/mTOR and RAS/RAF/MAPK pathways in HCC [49].

The abnormal expression of EGFR, VEGFR, and PDGFR also promotes HCC progression and metastasis through the PI3K/AKT/mTOR and RAS/RAF/MAPK pathways [50].

### 2.5. FGF19/CNND1

Approximately 5%–14% of HCC cases harbor FGF19/CNND1 alterations; their high expression is associated with poor prognosis [51]. It is now believed that FGFR4 is the sole receptor that displays specificity for FGF19/CNND1.

### 2.6. VEGFA

Based on fluorescence in situ hybridization, the estimated mutation rate of VEGFA in HCC is approximately 7%, and high VEGFA plasma levels are associated with poor prognosis in HCC [52,53]. VEGFA can promote tumor growth by stimulating angiogenesis and immune evasion.

## 3. Current Treatment Strategies for HCC

The treatment strategy for HCC is determined by the clinical stage. Despite the highest incidence of HCC occurring in the Asian region, BCLC prognosis and treatment strategy are not widely accepted because they do not align perfectly with the diagnostic and treatment characteristics of HCC, although BCLC prognosis and treatment strategy guidelines remain the most common consensus in Western countries. BCLC classifies HCC patients into five stages: BCLC-0 (very early), BCLC-A (early), BCLC-B (intermediate), BCLC-C (advanced), and BCLC-D (terminal). In BCLC-0- and BCLC-A-stage patients, curative methods such as hepatectomy, liver transplantation, and radiofrequency ablation are the main treatment methods. In intermediate HCC patients, local treatment, such as TACE, is the preferred treatment method. For advanced HCC patients, curative or local regional treatment is no longer suitable, and systemic therapy is a survival-benefit treatment option. For terminal-stage HCC patients, the best supportive care is recommended [14]. Corresponding to BCLC prognosis and treatment strategy, the estimated survival period for each stage is as follows: the early stage is more than 5 years, 2.5 years for the intermediate stage, 2 years for the advanced stage, and only 3 months for the terminal stage [14].

**Figure 3:**
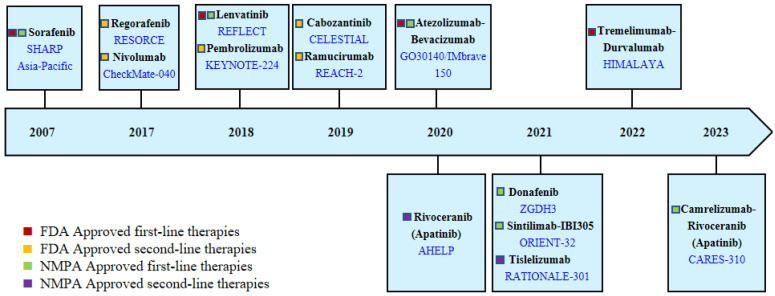
Therapies have been approved for advanced HCC by the Food and Drug Administration (FDA, USA) and the National Medical Products Administration (NMPA, China). This figure shows the therapies and corresponding clinical trials (blue font) that have been approved by the FDA and NMPA China to date.

Due to the insidious onset of symptoms, more than 50% of HCC patients diagnosed worldwide require systemic treatment [54]. In recent years, unlike the slow progress of curative approaches for early-stage HCC, systemic treatment regimens for advanced HCC have evolved rapidly. In 2007, the FDA approved Sorafenib for the treatment of advanced HCC, which significantly changed the treatment mode. In the following decade, many clinical trials were conducted, but these did not solicit much success. Until 2017, Regorafenib was approved as a second-line treatment for advanced HCC, and then the treatment of HCC entered a period of rapid development [55]. With the support of more successful clinical trials, more molecular-targeted and immunotherapy agents were approved for clinical use, as shown in Figure 3.

Despite the revolutionary advances that have been made in molecular-targeted therapy and immunotherapy for HCC, the overall survival of patients receiving systemic therapy is still limited, with only some patients benefiting from these therapies because of non-response and drug resistance. In this section, we will discuss approved systemic regimens that are commonly used in clinical practice.

### 3.1. Atezolizumab–Bevacizumab/Sintilimab–IBI305

For BCLC-C HCC patients having preserved liver function and no esophageal varices, the current guidelines recommend the use of Atezolizumab–Bevacizumab (PD-L1 inhibitor–VEGF inhibitor) as the first-line treatment [14].

Atezolizumab–Bevacizumab regimen is a new milestone in the field of HCC treatment. It is the first time that an immune checkpoint inhibitor in combination with antiangiogenic inhibitor drugs has been established as a first-line recommended regimen [7].

The combination of PD-1/PD-L1 inhibitors with VEGF inhibitors has been established as a new paradigm for the treatment of advanced HCC based on the IMbrave150 phase III clinical trial [44]. In advanced HCC patients with no prior treatment, the phase Ib study of Atezolizumab–Bevacizumab showed good safety and antitumor activity, with an achieved encouraging objective response rate (ORR) of 36% and median progression-free survival (mPFS) of 7.3 months [56]. Subsequently, in the Sorafenib-controlled IMbrave150 trial, Atezolizumab–Bevacizumab significantly reduced the risk of death and improved median overall survival (mOS), mPFS, and ORR [44] (details shown in Table 1).

The success of the Atezolizumab–Bevacizumab regimen may be attributed to synergistic antitumor activities. PD-L1 inhibition activates T cell immune response, while VEGF inhibition reduces VEGF-mediated immune suppression and enhances T cell function in the tumor microenvironment. Similar to the IMbrave150 trial, the ORIENT-32 trial tested Sintilimab (PD-1 inhibitor)–IBI305 (Bevacizumab biosimilar) versus Sorafenib in Chinese HCC patients. Compared to Sorafenib, Sintilimab–IBI305 showed significant improvements in terms of both mOS and mPFS (details shown in Table 2). Based on the ORIENT-32 trial, the National Medical Products Administration (NMPA) approved Sintilimab–IBI305 as a first-line treatment option in Chinese HCC patients [57].

Surprisingly, a recent clinical trial confirmed that Atezolizumab-Bevacizumab adjuvant therapy can reduce the recurrence in HCC patients who received curative treatment. The IMbrave050 clinical trial, which mainly included Asian HBV-HCC populations, showed that the risk of disease recurrence or death was 28% lower with Atezolizumab–Bevacizumab adjuvant treatment than with active surveillance (HR 0.72, adjusted 95% CI 0.53–0.98; *p* = 0.012). The difference in RFS event-free rates at 12 months was 13% (95% CI 6–20), while the median RFS was not reached in either group. IMbrave050 is the first positive phase 3 trial for adjuvant treatment in HCC, and it provides a new reference upon which to base further treatment advances for early-stage hepatocellular carcinoma in the future [15].

### 3.2. Tremelimumab–Durvalumab

Based on the results of the HIMALAYA phase III trial, the FDA approved Tremelimumab–Durvalumab (CTLA-4 inhibitor–PD-L1 inhibitor) for the first-line treatment of HCC in October 2022 [58]. The combination of Tremelimumab and Durvalumab is also a new milestone in the treatment of HCC as it represents the first recommendation for combination regimens of CTLA-4 inhibitor and PD-L1 inhibitor for the first-line treatment of HCC. Previously, the combination of PD-1/PD-L1 inhibitors and CTLA-4 inhibitors was the most studied immune–oncology combination in HCC. Theoretically, PD-1/PD-L1 inhibition enhances the antitumor activity of effector T cells, and CTLA-4 inhibition increases the abundance of CD4+ and CD8+ T cells in the tumor microenvironment, while the sufficient infiltration and adequate function of cytotoxic T lymphocytes are key to immunotherapy. The success of the HIMALAYA trial has translated this theory into clinical benefits [59].

The Nivolumab–Ipilimumab combination regimen is the first PD-1/PD-L1 inhibitor and CTLA-4 inhibitor combination regimen approved by the FDA for the second-line treatment of advanced HCC. A subset of the CheckMate 040 trial showed an impressive ORR of approximately 30% in patients previously treated with Sorafenib. Compared to immune checkpoint inhibitors monotherapy, Nivolumab–Ipilimumab combination therapy exhibited a higher response rate [60].

In the HIMALAYA phase III trial, Tremelimumab–Durvalumab combination regimens demonstrated significant efficacy as a first-line treatment. A new dosing approach called STRIDE (Single Tremelimumab Regular-Interval Durvalumab) was used in this study, which resulted in a statistically significant improvement in the STRIDE regimen compared to Sorafenib monotherapy. Additionally, the noninferiority of Durvalumab to Sorafenib was also demonstrated in this study [61,62] (details shown in Table 1).

Analysis of the data from the IMbrave150 and HIMALAYA trials suggests that Atezolizumab–Bevacizumab may be more effective than the STRIDE regimen, with a higher ORR (29.8% vs. 20.1%), better complete response rate (CR) (8% vs. 3%), and greater reduction in the risk of death (34% vs. 22%) compared to Sorafenib [62,63]. However, the HIMALAYA study showed that Tremelimumab–Durvalumab exhibited fewer treatment-related adverse events (TRAEs), such as no concern for gastrointestinal bleeding symptoms and no need for routine gastrointestinal endoscopy before administration. Nevertheless, it should be noted that the comparison between different clinical trials is less convincing/conclusive and more future assessments are needed (details shown in Table 3).

### 3.3. Sorafenib, Lenvatinib, Donafenib, and Rivoceranib

For BCLC-C HCC patients having preserved liver function who are not suitable for Atezolizumab–Bevacizumab or Tremelimumab–Durvalumab treatment, the current guidelines recommend systemic treatment with Lenvatinib/Sorafenib/Durvalumab [14]. Some guidelines suggest that if radiological progression is considered first rather than adverse events, Lenvatinib is preferred over Sorafenib [64].

Sorafenib was the first targeted therapy proven to be effective in advanced HCC, and it has been the standard of care for over a decade. The approval of Lenvatinib in 2018 further solidified the role of kinase inhibitors (KIs) in the first-line treatment of advanced HCC. In a preclinical study, Sorafenib showed better antitumor activity in TP53 wild-type HCC, while Lenvatinib is more sensitive in HCCs with TP53 mutation [65,66]. However, the therapeutic effects of both drugs are far from satisfactory. In HCC patients, Sorafenib only provides a survival benefit of 2.8 months compared to placebo [67]. Despite a high response rate, Lenvatinib has demonstrated noninferiority compared to Sorafenib and offers limited overall survival prolongation [68].

So far, in clinical trials and real-world studies, Lenvatinib has shown a tendency to be superior to Sorafenib, especially in HBV-related and AFP-elevated HCC subgroups [69,70].

Donafenib is a modified form of Sorafenib with improved pharmacokinetic characteristics. In the ZGDH3 phase III trial, Donafenib demonstrated a statistically significant improvement in overall survival compared to Sorafenib (details shown in Table 2) [71]. Based on the ZGDH3 phase III trial, the NMPA approved Donafenib as a first-line recommended drug in China. Rivoceranib (also known as Apatinib) is another KI with high selectivity for VEGFR2. In the AHELP phase III trial, compared to the placebo group, overall survival in the Rivoceranib group was significantly improved (details shown in Table 2) [72]. Based on the AHELP phase III trial, Rivoceranib was approved by the NMPA in 2020 for advanced HCC patients who have failed or are intolerant to at least one line of systemic treatment.

The same occurred with the ZGDH3 phase III trial. The limitation of the AHELP phase III trial is that the study population comes from a single geographic area.

### 3.4. Other Systemic Drugs

It is worth mentioning that based on the CARES-310 phase III trial, the NMPA approved the Camrelizumab–Rivoceranib (PD-1 inhibitor–VEGFR inhibitor) regimen as a first-line treatment for advanced HCC in China in 2023. In the CARES-310 phase III trial, Camrelizumab– Rivoceranib demonstrated excellent performance compared to Sorafenib, with an mOS of up to 22.1 months, the longest mOS among all systemic agents treated in HCC clinical trials so far. At the same time, the adverse events associated with Camrelizumab–Rivoceranib were manageable in this trial [73] (details shown in Table 2).

In the updated 2022 BCLC prognosis and treatment strategy recommendations, Regorafenib, Cabozantinib, and Ramucirumab are recommended as second-line treatments for patients who have progressed on Sorafenib [14]. It is believed that patients who switch to second-line treatment may benefit from Regorafenib if they tolerate Sorafenib; however, Regorafenib should not be used in patients who cannot tolerate Sorafenib due to toxicity. Ramucirumab may benefit patients with AFP levels >400 ng/dL who are resistant to Sorafenib. Cabozantinib may benefit patients who are tolerating Sorafenib, and it is also recommended as a third-line treatment [14]. Different from the NCCN guidelines of the United States, the prognosis and treatment strategy of the BCLC do not recommend Pembrolizumab, Nivolumab, or Ipilimumab due to insufficient supporting data.

For patients who fail first-line systemic therapy but still have preserved liver function, subsequent treatment can be selected comprehensively. Currently, there are no comparative studies among approved second-line therapies to guide the decision of the preferred regimen for second-line therapy.

## 4. The Challenges of Systemic Treatment for HCC

Since the launch of Sorafenib in 2007, the treatment paradigm of HCC has undergone revolutions. After 2017, based on more successful clinical trials, the application of new targeted and immunotherapy monotherapy or combination regimens has once again greatly advanced HCC treatment, prolonging the survival of patients. However, due to the high heterogeneity of HCC patients, less druggable mutation targets, and lack of effective biomarkers leading to inadequate stratification, HCC systemic treatment still faces many challenges.

### 4.1. The Lack of Effective Druggable Targets with a High Mutation Rate

The rationale of molecular-targeted therapy is to control tumor growth by inhibiting the molecular pathways that are essential for tumor growth and maintenance. Screening targets with a high mutation rate and effective intervention is the premise of molecular-targeted therapy. However, the precise molecular events leading to the formation of HCC are still only partially understood, and there are currently no satisfactory highly mutated and druggable targets. The main known mutation drivers of HCC, such as TERT, CTNNB1 (WNT/β-catenin), and TP53, are still considered undruggable [19,55]. Therefore, screening for highly mutated and druggable targets is the biggest challenge in the molecular-targeted therapy of HCC, and drugging the existing high mutation targets that have been discovered is also a challenging task, where new modalities are likely needed against interfering mutated genes.

Encouragingly, relevant research is ongoing. In a preclinical study of HCC, the silencing of TERT expression with antisense oligonucleotides achieved the inhibition of tumor growth in tumor cells and animal models [74]. Several inhibitors of the WNT/β-catenin pathway are currently being tested in clinical trials, such as targeted WNT inhibitors PRI-724 and BBI608 [75]. Accumulating lines of evidence also suggest that nonsteroidal anti-inflammatory agents (NSAIDs) such as celecoxib and sulindac can inhibit the WNT/β-catenin signaling pathway in human cancer cells [76]. The overexpression of wild-type AXIN1 can inhibit proliferation and accelerate the programmed cell death of HCC cell lines, indicating that AXIN1 is a therapeutic target in HCC if small molecules can enhance its expression and/or stability. Inhibiting poly-ADP-ribosylating enzymes tankyrase 1 and tankyrase 2 with small-molecule inhibitor XAV939 to stabilize AXIN is considered a new approach to targeting the WNT/β-catenin pathway [77]. In addition, drugs targeting upstream molecules of the WNT/β-catenin signaling pathway, such as salinomycin [78] and NVP-TNKS656 [79], have been developed and tested in preclinical HCC models.

Efforts targeting the p53 pathway are also underway. One study showed that small-molecule inhibitors of MDM4 may be effective against HCC [80], while arsenic trioxide potentially treats p53 mutant tumors by reactivating the mutated p53 protein [81].

### 4.2. Lack of Effective Biomarkers

Currently, systemic therapy for advanced HCC patients usually exhibits highly inconsistent efficacy, meaning that identifying biomarkers to predict drug efficacy and select appropriate treatment regimens is a matter of urgency. So far, HCC lacks effective molecular biomarkers to guide treatment [82].

It is generally believed that PD-1, PD-L1, CD3, and CD8 have a certain predictive effect on the efficacy of immune checkpoint inhibitors. Nevertheless, PD-L1, as a biomarker, is not reliable [83]. In NSCLC, up to 50% of patients with high PD-L1 expression do not respond to immune therapy [84]. In the CheckMate040 trial [60], Nivolumab had an objective response, regardless of tumor PD-L1 expression, whereas in KEYNOTE-224 [85], some patients responded to Pembrolizumab in association with PD-L1 expression. PD-L1 expression is regulated by multiple pathways, partly by the inherent expression of tumor cells, and partly by the release of IFN-γ when T cells kill tumor cells in the tumor microenvironment, which induces PD-L1 expression [86]. In addition, the expression of PD-L1 is influenced by various tumor signals, such as YAP/TAZ [87], which upregulates PD-L1 expression. The induction of PD-L1 expression by γ-IFN [88] has a good predictive effect on the efficacy of PD-1 inhibitors, while the relationship between PD-L1 from other pathways and the efficacy of PD-1 inhibitors is still unclear. This suggests that PD-L1 is not always a sufficient predictor of ICI therapy efficacy.

Pfister et al. conducted a study showing that anti-PD-1 therapy promotes the progression of NASH-induced HCC. Anti-PD-1 treatment expanded activated CD8+PD1+ T cells within the tumor; meanwhile, the incidence of NASH-HCC and the number and size of tumor nodules increased. Additionally, the increase in HCC induced by anti-PD1 therapy was prevented by either CD8+ T cell depletion or TNF neutralization. This study highlights once again that biomarker-based stratification of patients for optimal response to therapy is an unmet need [89].

Tumor mutation burden (TMB) is the most studied genomic biomarker and has demonstrated predictive ability in multiple tumors, such as melanoma, NSCLC, and bladder cancer [90]. However, one study claims that TMB did not show predictive ability in HCC [91].

There are also sporadic reports of biomarkers for KI drug selection, such as mutations in the PI3K-AKT-mTOR pathway being associated with the poor efficacy of Sorafenib, and VEGF, ANG2, and FGF21, as well as FGFR4 immunostaining positivity being associated with the clinical efficacy of Lenvatinib, but these approaches have not yet gained routine use in clinical practice as more comprehensive evaluations are needed [55].

Given the high heterogeneity of HCC, there is a lack of druggable targets with high mutation rates. A novel way of thinking about how to address HCC biomarkers in recent years is to look for the co-localization of biomarkers, leading to coherent molecular mechanisms.

Ankur Sharma et al. employed scRNA sequencing to extensively characterize the cellular landscape of the human liver from development to disease. They revealed a shared immunosuppressive oncofetal ecosystem in the fetal liver and HCC, shown as the enrichment of Tregs and exhausted CD8+T cells. Further results have suggested that VEGF and NOTCH signaling pathways play an important role in the maintenance of the immunosuppressive fetal cancer ecosystem [92]. Combined with the excellent performance of VEGF inhibitors in patients with HCC, the co-localization of biomarkers is more convincing.

### 4.3. How Do We Select Optimal Treatment Regimens Efficiently?

The high heterogeneity of HCC patients results in significant differences in response to the same agents among different patients. Current guidelines recommend diverse treatment regimens, which indeed increase the flexibility of clinicians, but also cause difficulties in choosing the right treatment for specific patients, resulting in randomness in therapy, a consequence not desired by the patients or doctors. For the selection of systemic agents for advanced HCC, currently, the most reliable evidence supports a sequential treatment, starting with Sorafenib. Depending on different situations, Regorafenib, Cabozantinib, and Ramucirumab can be chosen when the patient is progressing or becoming intolerant to Sorafenib, as mentioned above, although this is not the most ideal outcome for patients [14]. In this case, much more research is needed to identify biomarkers that are valuable in stratifying patients for response to different treatments.

Since more regimens have been recommended as first-line treatments, and because the performance of these new first-line regimens is better than, or at least not inferior to, Sorafenib, this undoubtedly reduces the probability of Sorafenib as the first-line option, which also leads to a lack of evidence supporting the selection of second-line treatment agents [14,93]. Again, more focused research to identify predictive biomarkers will be essential for better stratification of patients for the most suitable treatments.

More importantly, the selection among various first-line regimens currently lacks a strong scientific or clinical foundation, and the specific treatment for individual patients often requires comprehensive consideration of the first-line, second-line, and subsequent treatments. At present, there is insufficient evidence to support the selection of several regimens within the first-line treatment and the selection of various sequential treatments among the first-line and subsequent treatments. Therefore, a new therapy is desired that is superior to existing first-line therapies and is more applicable to an increased number of patients in addition to identifying predictable biomarkers for current therapies.

## 5. Potential Future Treatments

With the increasing progress in our understanding of the molecular mechanisms of HCC and future efforts in identifying predictive biomarkers for current therapies, we hope to transform the latest findings and knowledge into new targets, new therapies, and more precision biomarkers, ultimately improving patient outcomes. In fact, these discoveries have indeed contributed to the development of HCC treatment, such as immune checkpoint inhibitors becoming an important treatment, with a response rate of about 19% for anti-PD-1 therapy, including approximately 5% complete response and durable benefits in some patients [75]. However, despite the progress in HCC molecular therapy resulting from these discoveries, the effect on improving patient survival has been limited [93].

Based on the clinical performance of existing regimens, we believe that immunotherapy will remain the core of systemic treatment for HCC until new breakthrough therapies emerge. Various combination therapies and clinical trials focused on immunotherapy continue to drive the treatment progress made in HCC treatment. With an in-depth study on the tumorigenesis and drug resistance mechanism of HCC being revealed, precision therapy guided by molecular mechanisms, such as sequential combination therapy and synthetic lethality therapy, will be the future direction of HCC molecular treatment. In clinical practice, conversion therapy, which combines local therapy and systemic drugs as the method and aims to improve the R0 surgical resection rate of HCC patients, will also become an important area.

### 5.1. Exploration Surrounding Immunotherapy

The essence of immunotherapy is to activate the patient’s own immune system, especially for cytotoxic T cells to attack tumor cells. In HCC, the “inflammatory” subgroup accounts for about 30%–35% of all HCC patients, which is higher than most other tumor types. This inflammatory subgroup is sensitive to immunotherapy, which is why immunotherapy has become the main systemic treatment for HCC [54]. As mentioned above, the most commonly used immune regimens in HCC include dual-immunotherapy combination therapy, the combination of ICIs and anti-VEGF/VEGFR, and combinations of ICIs and KIs. Additionally, other immunotherapy approaches are also being evaluated in HCC.

#### 5.1.1. Dual-Immunotherapy Combination Therapy

The mechanisms of different antitumor immune inhibitors vary greatly, providing opportunities for developing dual or multiple immunotherapy combinations. Currently, PD-1/PD-L1 inhibitor and CTLA-4 inhibitor combination regimens are the most studied immune combination in HCC. Tremelimumab (CTLA-4 inhibitor)–Durvalumab (PD-L1 inhibitor) combination therapy and Nivolumab (PD-1 inhibitor)–Ipilimumab (CTLA-4 inhibitor) combination therapy have been approved by the FDA as the first-line and second-line treatment of advanced HCC, respectively [14]. Some studies suggest that PD-1 inhibitors may provide better mOS and mPFS than PD-L1 inhibitors because they can simultaneously block PD-L1 and PD-L2; however, more clinical trials are needed to support this hypothesis [94]. Another phase III clinical trial of dual-immunotherapy combination evaluating the combination of Nivolumab–Ipilimumab for the first-line treatment of HCC, CheckMate 9DW, is currently ongoing, with Sorafenib/Lenvatinib used as the control [55,95]. In the future, there should be more combination therapies of PD-1/PD-L1 inhibitors and CTLA-4 inhibitors entering clinical use with the support of clinical trials.

#### 5.1.2. Combination of ICIs and Anti-VEGF/VEGFR

Aberrant tumor angiogenesis driven by VEGF can lead to the formation of an immunosuppressive tumor microenvironment. VEGF/VEGFR inhibitors can inhibit angiogenesis and reprogram the tumor microenvironment through various mechanisms [96]. The Atezolizumab (PD-L1 inhibitor)–Bevacizumab (VEGF inhibitor) regimen and the Sintilimab (PD-1 inhibitor)–IBI305 (VEGF inhibitor) regimen have been approved by the FDA and NMPA for the first-line treatment of advanced HCC, respectively [55,97].

In 2023, the NMPA approved the Camrelizumab (PD-1 inhibitor)–Rivoceranib (VEGFR inhibitor) regimen as a first-line treatment for advanced HCC in China. In the CARES-310 phase III trial, the Camrelizumab–Rivoceranib combination regimen is significantly superior to Sorafenib in terms of both mOS and mPFS, and mOS reached an unprecedented 22.1 months, demonstrating the good prospects of the combination of ICIs and anti-VEGF/VEGFR regimens [73] (details shown in Table 3).

In addition, the safety and preliminary efficacy of the Durvalumab–Ramucirumab combination regimen has also been evaluated in a phase Ia/b JVDJ trial [98], which comprised various solid tumors, including HCC. Ramucirumab may be safer than Bevacizumab [55,99].

#### 5.1.3. Combinations of ICIs and KIs

Kis not only target the corresponding tumor signaling pathways but also can have an antiangiogenic effect. Compared with antiangiogenic drugs, they usually have relatively stronger tumor-killing and tumor necrosis activity, thus inducing the release of more tumor antigens and enhancing tumor immunogenicity [55]. In addition, because their targets include VEGFRs, they can also exert immunomodulatory functions similar to VEGF antibodies, which is more conducive to being synergistic with ICIs [100]. Therefore, theoretically, the combination of ICIs and Kis is one of the most promising combination regimens.

Almost all Kis currently used in clinical practice have been evaluated for their synergistic potential with ICIs, among which Lenvatinib is the most studied drug. In the previous KEYNOTE-524 phase I study, the ORR of Pembrolizumab (PD-1 inhibitor)–Lenvatinib (tyrosine kinase inhibitor) reached 36.0% [101,102], which was double that achieved with Pembrolizumab monotherapy. Therefore, the results of the LEAP-002 trial evaluating the Pembrolizumab–Lenvatinib regimen versus Lenvatinib monotherapy were highly anticipated. However, the LEAP-002, announced in 2022, showed that the primary endpoints of mOS and mPFS did not reach prespecified statistical significance [103]. Nevertheless, the data showed that the Pembrolizumab–Lenvatinib combination achieved a very satisfactory mOS (21.2 months), and no new severe TRAEs were observed [103] (details shown in Table 3).

At present, the two recommended regimens for the first-line systemic treatment of HCC are Atezolizumab–Bevacizumab and Tremelimumab–Durvalumab. In updated Imbrave-150 data, the mOS of Atezolizumab–Bevacizumab is 19.2 months [63]. Tremelimumab–Durvalumab showed a mOS of 16.4 months in the HIMALAYA trial study [59]. In some subgroup analyses, the Pembrolizumab–Lenvatinib combination demonstrated a significant advantage, with a 22% decrease in death risk for the subgroup with macrovascular invasion/extrahepatic spread and a 33% decrease in death risk for the subgroup with elevated AFP [103]. Additionally, LEAP-002 used Lenvatinib as the control agent, while almost all phase III clinical trials to date have used Sorafenib as the control. If Sorafenib was also used as a control in this trial, the results would be of interest (details shown in Table 3).

Recently, the results from a real-world study in China support the efficacy of the Pembrolizumab–Lenvatinib regimen in advanced HCC. In this real-world study, which included 378 patients with unresectable HCC, 89.9% of cases were caused by HBV infection. The mOS for the Pembrolizumab–Lenvatinib regimen was 17.8 months, the median mPFS was 6.9 months, and the ORR and DCR were 19.6% and 73.5%, respectively. The researchers concluded that the Pembrolizumab–Lenvatinib regimen showed promising survival, ORR, and DCR in real-world studies [104]. Increasingly, clinical trial data for targeted and immune therapies have shown that a high ORR does not always translate into prolonged overall survival. Many other trials combining Lenvatinib with other ICIs are also underway, and their results are also eagerly awaited [55].

There are many other clinical trials of ICIs combined with Kis, such as Axitinib and Anlotinib, showing promising preliminary results, with ORRs as high as 30%. Although no ICI-KI combined regimen has been approved for the clinical treatment of HCC, it is likely to be one of the most promising regimens.

#### 5.1.4. Other Immunotherapy

In addition to the immunotherapy strategies mentioned above that have been extensively studied, many other immunotherapies are being tested for HCC, such as CAR-T cell therapy, which aims to increase the T cell infiltration of engineered T cells [105], TGFβ inhibitors that relieve immune-suppressive signals in the tumor microenvironment [106], PRL3–Zumab [107], therapeutic vaccines [108], and bispecific antibodies [109], etc.

In regards to the development of targeted agents against other immune modulatory molecules, these drugs can synergize and complement the effects of PD-1/PD-L1 or CTLA-4 inhibitors to treat tumors [94]. In 2022, the combination of the LAG3 inhibitor Relatlimumab and Nivolumab was approved by the FDA for the treatment of melanoma [110]. At the same time, other regimens, including Relatlimumab–Nivolumab, which is different from PD-1/PD-L1 or CTLA-4 inhibitors, are also actively undergoing clinical research for the treatment of HCC.

Furthermore, in recent years, increasing research has detected a close relationship between the Hippo signaling pathway and immunotherapy [111]. The main effectors of the Hippo pathway, YAP/TAZ, are associated with PD-L1 expression in various tumors [87]. YAP/TAZ are found to be highly expressed in HCC cells, MDSCs, and Tregs, and are closely related to immunotherapy [112,113,114]. Several studies suggest that targeting YAP/TAZ can significantly downregulate the function of immune-inhibitory cells in the tumor microenvironment, and some preclinical trials have been confirmed [87]. The role of the Hippo signaling pathway in immunotherapy requires further in-depth investigation.

Enhanced toxicity is a major challenge when using combination therapy. Current evidence suggests that the dual-immunotherapy combination appears to have the best safety in HCC, followed by a combination of ICIs and anti-VEGF/VEGFR, and then combinations of ICIs and KIs [55].

### 5.2. Precision Therapy Guided by Molecular Mechanisms

Along with our further understanding of HCC tumorigenesis, some combination therapies based on molecular mechanisms have arisen, such as sequential combination therapy and synthetic lethality therapy, which can be defined as precision therapy guided by molecular mechanisms.

Sequential combination therapy is a targeted therapy strategy that has emerged in the past few years. The basic strategy is to administer drugs one after another, with the first drug inducing the vulnerability of cancer cells, making them more susceptible to the second drug, increasing the synergistic antitumor effect while reducing the toxicity of the combination. The STRIDE regimen used in the HIMALAYA trial is a good example of sequential administration [59]. In this trial, a single dose of 300 mg Tremelimumab was given as a starting dose, followed by regular intervals of Durvalumab. The starting dose of Tremelimumab can improve the abundance and function of T cells in the tumor microenvironment by inhibiting CTLA-4, while Durvalumab works by inhibiting PD-L1 to activate T cells to exert an antitumor effect, thereby showing synergistic effects.

The activation of compensatory signaling pathways is a major reason for molecular-targeted therapy resistance. Based on an understanding of the molecular mechanisms of compensatory signaling pathways, inhibiting the activated bypass pathway to achieve the reversal of drug resistance is the main strategy used to overcome molecular-targeted drug resistance. Studies have found that after treatment with Lenvatinib for HCC, the expression of EGFR in tumor tissue increases. Mechanistically, Lenvatinib treatment inhibits FGFR, leading to feedback activation of the EGFR-PAK2-ERK5 signaling axis. Researchers have combined the EGFR inhibitor Gefitinib with Lenvatinib for EGFR-High expression HCC treatment. This combination regimen achieves meaningful clinical responses in 12 advanced HCC patients unresponsive to Lenvatinib monotherapy [115].

Interestingly, in the SEARCH phase III study without EGFR expression prescreening, the combination of Sorafenib and EGFR inhibitor did not show a statistically significant efficacy improvement [116]. The contrasting results of these two studies highlight the importance of thoroughly studying the molecular mechanisms of treatment.

The EMERALD-1 phase III clinical trial, announced in 2024, explored the efficacy of TACE in combination with Durvalumab, with or without Bevacizumab, against patients with embolization-eligible uHCC. At the final analysis, mPFS significantly improved for Durvalumab + Bevacizumab +TACE vs TACE (15.0 vs. 8.2; HR 0.77; *p* = 0.032). The safety was manageable and consistent with the safety of Durvalumab, Bevacizumab, and TACE in uHCC.

It is worth mentioning that the background of the EMERALD-1 design is that TACE generates proinflammatory tumor microenvironment and increases VEGF signaling, leading to most people with uHCC treated with TACE progress within 1 year. The positive results of the EMERALD-1 trial also underscore Precision therapy guided by molecular mechanisms [117].

### 5.3. Conversion Therapy Aimed at Improving the R0 Resection Rate

Although systemic drugs have been the fastest-growing field in HCC treatment for over a decade, radical surgical resection remains the best treatment because it can provide the longest survival period or even a cure. Based on the background of the rapid development of systemic treatment agents, conversion therapy aimed at improving the R0 resection rate of HCC patients has become a hot topic for clinicians, and it has achieved encouraging results [118].

The goal of conversion therapy is to transform unresectable advanced HCC into the resectable stage. Commonly used conversion therapies include local treatment (TACE, transarterial radioembolization, or hepatic arterial infusion chemotherapy (HAIC)), systemic treatment (targeted therapy alone or combined with immunotherapy), and a therapeutic alliance (TACE combined with radiotherapy, TACE combined with targeted therapy, HAIC combined with targeted therapy, or HAIC combined with targeted therapy and immunotherapy) [119].

In a study of 63 patients with unresectable HCC, combination therapy with KI and anti-PD-1 antibodies was used for treatment, and 10 patients underwent R0 resection surgery 3.2 months later. Postoperatively, one patient died from immune-related adverse events 2.4 months after hepatectomy. After a median follow-up of 11.2 months in the other nine patients, eight patients remained alive without disease recurrence, and one suffered tumor recurrence [120]. This study indicates that the combination of KI and PD-1 antibodies is a feasible conversion therapy for patients with unresectable HCC.

There have been many successful conversion therapy reports. In the IMbrave150 trial, the Atezolizumab–Bevacizumab regimen demonstrated excellent antitumor activity against tumor invasion in the main portal trunk (Vp4). Vascular invasion is one of the main reasons why HCC patients are not suitable for R0 resection. Therefore, the IMbrave150 trial has also sparked the expectation for the use of the Atezolizumab–Bevacizumab regimen in conversion therapy [121].

It is worth noting that, with the continuous increase of HCC treatment approaches, the management of HCC patients has become more complex. The current treatment strategy based strictly on stage is being challenged. Recently, the Italian Association for the Study of the Liver put forward a new concept of HCC treatment, that is, to choose a more personalized treatment plan for patients with survival-benefit as the orientation, and expert tumor boards should assume a central role in the selection of treatment [122].

## 6. Conclusions

Although HBV and HCV remain the most common risk factors for HCC worldwide, the proportion of metabolic-related HCC is increasing. In recent years, the treatment of HCC has achieved rapid development, mainly manifested in an increasing number of targeted and immunological agents approved for systemic treatment, as well as the establishment of immunotherapy as the standard of care. The prospect of HCC treatment in the short term includes the continuous emergence of various combination therapies centered around immunotherapy agents, the development of a precision dosing regimen guided by exact molecular mechanisms, and the success of conversion therapy under the combination of local and systemic treatments. Currently, the treatment of HCC still faces many challenges, such as the lack of effective druggable targets with high mutation, the lack of effective molecular markers for patient stratification and treatment guidance, and the lack of efficient guidelines for selecting optimal treatment. The solutions to the above issues will depend on further research into the molecular mechanisms of HCC and the identification and application of predictive biomarkers.

## Figures and Tables

**Table 1:** The critical results of phase III clinical trials for therapies approved by the FDA.

Treatments (Trial)	Dosing Regimen	Line Setting/ Patients Number	Outcome				TRAE		Remark
			mOS (Months) (HR, *p*-Value)	mPFS (Months) (HR, *p*-Value)	ORR (%)	CR (%)	≥Grade 3 (%)	Death/Grade 5 (%)	
Sorafenib vs. Placebo (SHARP)	KI monotherapy	1L/602	10.7 vs. 7.9 HR 0.69, *p* < 0.001	NA	NA	NA	NA	NA	TTP: 5.5 vs. 2.8 months (HR 0.58, *p* < 0.001); any-grade TRAEs: 80% vs. 52%
Sorafenib vs. Placebo (Asia–Pacific)	KI monotherapy	1L/271	6.5 vs. 4.2 HR 0.68, *p* = 0.014	NA	3.3 vs. 1.3 Based on RECIST v1.0	NA	NA	NA	TTP: 2.8 vs. 1.4 months (HR 0.57, *p* = 0.0005)
Lenvatinib vs. Sorafenib (REFLECT)	KI monotherapy	1L/954	13.6 vs. 12.3 HR 0.92, noninferiority Based on mRECIST	7.3 vs. 3.6 HR 0.65, *p* < 0.0001	18.8 vs. 6.5	0.4 vs. <0.2	56.7 vs. 48.6	2.3 vs. 0.8	The mOS of Lenvatinib is noninferior to Sorafenib in the REFLECT trial. However, Lenvatinib has shown a tendency to be superior to Sorafenib, especially in HBV-related and AFP-elevated HCC subgroups.
Regorafenib vs. Placebo (RESORCE)	KI monotherapy	2L/567	10.6 vs. 7.8 HR 0.63, *p* < 0.0001	3.4 vs. 1.5 HR 0.43, *p* < 0.0001	6.6 vs. 2.6	0.5 vs. 0	50.0 vs. 16.6	1.9 vs. 1.0	
Cabozantinib vs. Placebo (CELESTIAL)	KI monotherapy	2L/707	10.2 vs. 8.0 HR 0.76, *p* = 0.005	5.2 vs. 1.9 HR 0.44, *p* < 0.001	3.8 vs. <0.4	0 vs. 0	67.7 vs. 36.3	1.3 vs. 0.4	
Ramucirumab vs. Placebo (REACH-2)	anti-VEGFR monotherapy	2L/292	8.5 vs. 7.3 HR 0.71, *p* = 0.0199	2.8 vs. 1.6 HR: 0.45, *p* < 0.0001	4.6 vs. 1.1	NA	NA	1.% vs. 0	Only recommended for patients with AFP ≥ 400 ng/mL.
Nivolumab vs. Sorafenib (CheckMate459)	anti-PD-1 monotherapy	1L/743	16.4 vs. 14.7 HR: 0.85, *p* = 0.075	3.7 vs. 3.8 HR:0.93 *p* value NA	15.4 vs. 6.9	3.8 vs. 1.3	22.3 vs. 49.6	NA	Based on CheckMate 040, Nivolumab was approved by the FDA as a second-line treatment for advanced HCC. However, the indication of Nivolumab as a monotherapy for second-line treatment of advanced HCC has been withdrawn due to the failure of CheckMate 459.
Pembrolizumab vs. Sorafenib (KEYNOTE-394)	anti-PD-1 monotherapy	2L/453	14.6 vs. 13.0 HR 0.79, *p* = 0.0180	2.6 vs. 2.3 HR 0.74, *p* = 0.0032	13.7 vs. 1.3	2.0 vs. 0.7	14.4 vs. 5.9	1.0 vs. 0	Pembrolizumab was approved by the FDA for advanced HCC second-line treatments based on KEYNOTE-224, while KEYNOTE-394 is the updated support trial.
Atezolizumab–Bevacizumab Vs. Sorafenib (IMbrave150)	anti-PD-L1 and anti-VEGF	1L/501	19.2 vs. 13.4 HR 0.66, *p* < 0.001	6.9 vs. 4.3 HR 0.65, *p* < 0.001	29.8 vs. 11.3	7.7 vs. 0.6	43.5 vs. 46.2	1.8 vs. 0.6	Patients should have adequate endoscopic evaluation and management for esophageal varices before administration.
Tremelimumab–Durvalumab vs. Sorafenib (HIMALAYA)	anti-CTLA-4 and anti-PD-L1	1L/1171	16.4 vs. 13.8 HR: 0.78, *p* = 0.0035	3.8 vs. 4.1 HR:0.90, *p* value NA	20.1 vs. 5.1	3.1 vs. 0	25.8 vs. 36.9	2.3 vs. 0.8	
Durvalumab vs. Sorafenib (HIMALAYA)	anti-PD-L1 monotherapy	1L/1171	16.6 vs. 13.8 HR:0.86, noninferiority margin 1.08	3.7 vs. 4.1 HR:1.02, *p* value NA	17.0 vs. 5.1	1.5 vs. 0	12.9 vs. 36.9	0 vs. 0.8	

Objective response rate (ORR) and progression-free survival (PFS) are based on RECIST version 1.1 unless otherwise indicated. TRAEs, treatment-related adverse events; mOS, median overall survival; mPFS, median progression-free survival; ORR, objective response rate; CR, complete response rate; KI, kinase inhibitors; 1L, first-line; 2L, second-line; NA, not available; TTP, time to progression; HR, hazard ratio;

**Table 2:** The critical results of phase III clinical trials for therapies approved by the NMPA *.

Treatments (Trial)	Dosing Regimen	Line Setting/ Patients Number	Outcome				TRAE		Remark
			mOS (Months) (HR, *p* Value)	mPFS (Months) (HR, *p* Value)	ORR (%)	CR (%)	≥Grade 3 (%)	Death/Grade 5 (%)	
Rivoceranib vs. Placebo (AHELP)	anti-VEGFR monotherapy	2L/400	8.7 vs. 6.8 HR 0.785, *p* = 0·048	4.5 vs. 1.9 HR 0·471, *p* <0·0001;	10.7 vs. 1.5	0 vs. 0	77.4 vs. 19.2	0 vs. 0	The study population is limited to China
Donafenib vs. Sorafenib (ZGDH3)	KI monotherapy	1L/668	12.1 vs. 10.3 HR 0.83, *p* = 0.0245	3.7 vs. 3.6 HR 0.91, *p* = 0.057	4.6 vs. 2.7	0.3 vs. 0	37.5 vs. 49.7	1.8 vs. 3.6	The study population is limited to China
Tislelizumab vs. Sorafenib (RATIONALE-301)	anti-PD-1 monotherapy	1L/674	15.9 vs. 14.1 HR: 0.85, *p* = 0.0398	2.2 vs. 3.6 HR: 1.11, *p* value NA	14.3 vs. 5.4	2.9 vs. 0.3	22.2 vs. 53.4	4.4 vs. 5.2	
Sintilimab + IBI305 vs. Sorafenib (ORIENT-32)	anti-PD-1 and anti-VEGF	1L/571	NE vs. 10.4 HR 0.57, *p* < 0.0001	4.6 vs. 2.8 HR 0.56, *p* < 0.0001	20.5 vs. 4.1	0 vs. 0	33.7 vs. 35.7	1.6 vs. 1.0	The study population is limited to China
Camrelizumab–Rivoceranib vs. Sorafenib (CARES-310)	anti-PD-1 and anti-VEGFR	1L/543	22.1 vs. 15.2 HR 0.62, *p* < 0.0001	5.6 vs. 3.7 HR 0.52, *p* < 0.0001	25.4 vs. 5.9	1.1 vs. 0.4	80.9 vs. 52.4	0.4 vs. 0.4	

Objective response rate (ORR) and progression-free survival (PFS) are based on RECIST version 1.1 unless otherwise indicated. TRAEs, treatment-related adverse events; mOS, median overall survival; mPFS, median progression-free survival; ORR, objective response rate; CR, complete response rate; KI, kinase inhibitors; 1L, first-line; 2L, second-line; NA, not available; HR, hazard ratio; NE, not estimable. * This table only includes NMPA-approved treatment therapies for advanced HCC developed by Chinese companies.

**Table 3:** Comparison of phase III clinical trials of different immunotherapy combination strategies.

		(HIMALAYA)	(IMbrave150)	(CARES-310)	LEAP 002
Immunotherapy Combination Strategies		Dual-Immunotherapy (Anti-CTLA-4 and Anti-PD-L1)	ICIs and Anti-VEGF/VEGFR (Anti-PD-L1 and Anti-VEGF)	ICIs and Anti-VEGF/VEGFR (Anti-PD-1 and Anti-VEGFR)	ICIs and KIs (Anti-PD-1 And KI)
Treatments		Tremelimumab–Durvalumab	Atezolizumab–Bevacizumab	Camrelizumab–Rivoceranib	Pembrolizumab–Lenvatinib
Control agent		Sorafenib	Sorafenib	Sorafenib	Lenvatinib
Endpoints and results (RECIST 1.1)		Primary endpoint: mOS STRIDE arm was superior to Sorafenib; Durvalumab monotherapy was noninferior to Sorafenib.	Primary endpoint: mOS and mPFS Both meet with statistical significance.	Primary endpoint: mOS and mPFS Both meet with statistical significance.	Primary endpoint: mOS and mPFS Neither meets the prespecified statistical significance.
Outcome	mOS (months) (HR, *p* value)	STRIDE vs. Sorafenib: 16.43 vs. 13.77 HR: 0.78, *p* = 0.0035 Durvalumab vs. Sorafenib: 16.56 vs. 13.77 HR:0.86, noninferiority margin 1.08	19.2 vs. 13.4 HR: 0.66 *p* = 0.0009	22.1 vs. 15.2 HR: 0.62 *p* < 0.0001	21.2 vs. 19.0 HR: 0.840 *p* = 0.0227
	mPFS (months) (HR, *p* value)	STRIDE vs. Durvalumab vs. Sorafenib: 3.78 vs. 3.65 vs. 4.07 *p* value NA	6.9 vs. 4.3 HR: 0.65 *p* = 0.0001	5.6 vs. 3.7 HR: 0.52 *p* < 0.0001	8.2 vs. 8.0 HR: 0.867 *p* = 0.0446
	ORR (%)	STRIDE vs. Durvalumab vs. Sorafenib: 20.1 vs. 17.0 vs. 5.1	29.8 vs. 11.3	25.4 vs. 5.9	26.1 vs. 17.5
	CR (%)	STRIDE vs. Durvalumab vs. Sorafenib: 3.1 vs. 1.5 vs. 0	7.7 vs. 0.6	1.1 vs. 0.4	1.5 vs. 1.5
	PR (%)	STRIDE vs. Durvalumab vs. Sorafenib: 17.0 vs. 15.4 vs. 5.1	22.1 vs. 10.7	24.3 vs. 5.5	24.6 vs. 16.0
	SD (%)	STRIDE vs. Durvalumab vs. Sorafenib: 39.9 vs. 37.8 vs. 55.5	44.2 vs. 43.4	52.9 vs. 48.0	55.2 vs. 60.9
	DCR (%)	STRIDE vs. Durvalumab vs. Sorafenib 60.1 vs. 54.8 vs. 60.7	73.9 vs. 54.7	78.3 vs. 53.9	81.3 vs. 78.4
	DoR (months)	STRIDE:22.34 STRIDE: long-tail effect; 3-year survival rate was 30.7%	18.1	14.8	16.6
Safety profile	TRAE ≥Grade 3 (%)	STRIDE vs. Durvalumab vs. Sorafenib: 25.8 vs. 12.9 vs. 36.9	43.5 vs. 46.2	80.9 vs. 52.4	61.5 vs. 56.7
	Discontinuation (%)	STRIDE vs. Durvalumab vs. Sorafenib: 8.2 vs. 4.1 vs. 11.0	15.5 vs. 10.3	24.3 vs. 4.5	18.0 vs. 10.6
	TRAE Grade 5/Death (%)	STRIDE vs. Durvalumab vs. Sorafenib: 2.3 vs. 0 vs. 0.8	1.8 vs. 0.6	0.4 vs. 0.4	1.0 vs. 0.8
Remark		The success of the HIMALAYA trial transformed the theoretical advantages of dual-immunotherapy treatment into clinical benefits. STRIDE regimen is a successful paradigm of sequential combination therapy. Dual-immunotherapy treatment shows an excellent DoR, a long-tail effect, and lower toxicity.	The IMbrave150 trial established the anti-PD-L1/PD-1 inhibitor and anti-VEGF/VEGFR combination strategies as the first-line recommendation for advanced HCC. Atezolizumab–Bevacizumab combination therapy significantly improved mOS of patients with portal vein invasion at the main portal branch (Vp4).	The CARES-310 trial reached the longest mOS in clinical trials for advanced HCC systemic treatment agents. Comparison of HR between trials shows anti-PD-1/PD-L1 and TKI/anti-VEGF have better ORR and PD outcomes than dual-immunotherapy regimens.	The LEAP 002 trial is the sole clinical trial with a double-blind design in this table, and it is the sole clinical trial that used Lenvatinib as the control agent in this table. Pembrolizumab–Lenvatinib therapy shows a clear tendency of OS benefit, although the prespecified significance endpoint is not met.

Objective response rate (ORR) and progression-free survival (PFS) are based on RECIST version 1.1 unless otherwise indicated. mOS, median overall survival; mPFS, median progression-free survival; ORR, objective response rate; CR, complete response rate; PR, partial response; SD, stable disease; DCR, disease control rate; DoR, duration of response; STRIDE, single Tremelimumab regular-interval Durvalumab; HR, hazard ratio.
